# Prevalence of post-traumatic stress disorder in Peruvian military: a cross-sectional study

**DOI:** 10.3389/fpsyt.2025.1573379

**Published:** 2025-10-22

**Authors:** Mario J. Valladares-Garrido, Cinthia Karina Picón-Reátegui, J. Pierre Zila-Velasque, Virgilio E. Failoc-Rojas, César Johan Pereira Victorio, Danai Valladares-Garrido, Víctor J. Vera-Ponce

**Affiliations:** ^1^ Escuela de Medicina Humana, Universidad Señor de Sipán, Chiclayo, Peru; ^2^ Faculty of Medicine, Universidad de San Martín de Porres, Chiclayo, Peru; ^3^ Red Latinoamericana de Medicina en la Altitud e Investigación (REDLAMAI), Pasco, Peru; ^4^ Research Unit for Generation and Synthesis Evidence in Health, Universidad San Ignacio de Loyola, Lima, Peru; ^5^ School of Medicine, Universidad Continental, Lima, Peru; ^6^ Escuela de Medicina, Universidad César Vallejo, Piura, Peru; ^7^ Oficina de Salud Ocupacional, Hospital de Apoyo II Santa Rosa, Piura, Peru; ^8^ Facultad de Medicina (FAMED), Universidad Nacional Toribio Rodríguez de Mendoza de Amazonas (UNTRM), Amazonas, Peru

**Keywords:** PTSD, mental health, Latin Americans, COVID-19, military

## Abstract

**Introduction:**

The COVID-19 pandemic has led us to stay at home in order to mitigate the increase in contagion, which has modified military personnel’s work as they had to be on the front-line of the global fight. Post-traumatic stress disorder (PTSD) is a psychiatric condition that has become a challenge for public health. Little research has been undertaken in military population, even less in the Latin American context. The objective is determining the prevalence and factors associated with post-traumatic stress disorder in military personnel in Lambayeque, Peru.

**Methods:**

This is an analytic cross-sectional observational study in military staff that carried out first-line activities on the defense against COVID-19. The study population was comprised of 820 military personnel. To evaluate the factors associated with PTSD, we calculated prevalence ratios (PR) and confidence intervals at 95%, using simple and multiple regression models.

**Results:**

The prevalence of PTSD was 7.8% (95% CI: 5.8%–10.2%). The multiple regression model showed that smoking (PR: 2.84; 95% IC: 1.14-7.09), having worked between 13 and 18 months (PR: 2.62; 95% IC: 1.20-5.75), insomnia (PR: 4.09; 95% IC: 1.90-8.83), and fear of COVID (PR: 6.20; 95% IC: 2.70-14.22) were associated with a higher prevalence of PTSD in military personnel.

**Conclusion:**

We found that nearly one in ten military personnel presented PTSD. Factors associated with a higher prevalence included longer service time, smoking, insomnia, and fear of COVID-19. Although resilience showed a protective trend in crude analyses, this association was not significant after adjustment.

## Introduction

The COVID-19 pandemic has caused negative repercussions for mental health worldwide (1–7). Among these negative consequences, we can highlight post-traumatic stress disorder (PTSD), which emerges after exposure to a severe trauma or life-threatening event such as the COVID-19 pandemic. PTSD occurs when the nervous system remains in a persistent state of stress activation, meaning that although the immediate danger has passed, the individual continues to experience heightened arousal and is unable to fully return to baseline functioning (8).

The pandemic has made us stay at home to mitigate the increase in contagion through quarantines and isolation, which have modified work for military personnel who stood in the front-line of defense worldwide to ensure the compliance of the mentioned measures (9). Among the factors associated with the highest prevalence of PTSD in military members, we can mention bad sleep quality, a low level of social support (10–13), being an older adult (10, 14), lower official ranks (10, 12, 14). On the other hand, among the factors associated with low prevalence we can mention a long time of service (14), and having a high level of social network support (15).

According to current literature, PTSD is a common psychiatric condition in military personnel worldwide and has become a challenge for public health (16). However, the prevalence reported before the pandemic ranged from 3.0% to 30.0% in countries such as the United States, United Kingdom, Canada, and the Netherlands (17–20). At Latin American level it has been reported that, in Colombia, the prevalence of PTSD varies between 4.90% and 36.0% (21, 22); in Mexico, 4.3% (23); in Peru, 22% (24); and Chile, 15.0% (25). This situation differs from the studies conducted in the pandemic since they have reported PTSD prevalence from 12.8% to 31.2% among active military personnel and veterans (15, 26, 27). However, the existent literature has focused on other realities different from Latin America such as in veterans (military personnel that do not have deployment activity on the first line of defense against COVID-19) (26–29), or other population groups such as health personnel, general population, or infected patients (30–33).

In addition, there have been limitations identified in some studies such as not having measured variables such as uncertainty intolerance or income level (34). In other studies, a four-item instrument which do not have a high reliability was used (35); data collection was performed online (36); the sample size used was small (22, 37); the studied populations corresponded to veterans (not active military personnel) (38, 39); thus, the results cannot be generalized in groups with an active status. Additionally, the studies conducted during the pandemic have not evaluated variables such as comorbidities (40), mental health support (41), time of service, food insecurity (42), or resilience (43), which were evaluated in our study.

Therefore, our study aims to identify the prevalence and factors associated with post-traumatic stress disorder in Peruvian military personnel on the first line of defense in the context of the pandemic.

## Methods

### Study design

This is an analytic cross-sectional observational study conducted in military staff that carried out front-line defense activities against COVID-19 in the infantry brigade in Lambayeque, Peru. The study was undertaken between November 02 to 09, with the objective of assessing the prevalence and factors associated with post-traumatic stress disorder.

### Population and sample

The study population was comprised of 820 military personnel of the infantry brigade located in the region of Lambayeque, Peru. This region is in the north of Peru and was severely hit by the COVID-19 pandemic as it showed a high level of seroprevalence (44) and mortality (45) due to SARS-CoV-2.

We calculated a sample size of 582 participants. The study used an expected prevalence of 12.8%, a confidence level at 99%, a precision of 2.5% and a rejection and loss rate of 20%. However, we obtained a higher participation in the execution (n=710 military members) and the sample gave their consent to participate in the study, which represented 86.6% of the population. We applied a snowball sampling strategy. Initially, the survey link was distributed to military supervisors, who subsequently shared it with other eligible members of their units. Although snowball sampling is a non-probability method and carries a risk of selection bias, it was the only feasible strategy during the COVID-19 state of emergency due to operational restrictions and the hierarchical structure of the military. To reduce this potential bias, recruitment was coordinated directly with unit supervisors, and coverage was maximized by including personnel from all service groups within the brigade.

The military members included were actively working during the COVID-19 health emergency in Lambayeque when they participated in the study and had at least one month of service. The military personnel excluded were the following: those who requested temporary layoff due to factors of risk of COVID-19, those vulnerable military members that were working remotely, and those who were isolated for having tested positive for COVID-19. Additionally, for this analysis, we excluded 95 participants for having incomplete variables in questions of the PCL-C questionnaire, given that it measures the dependent variable. Thus, we analyzed 615 military records.

### Variables and instruments

They have seven sections that covered 1) sociodemographic data, 2) Household Food Insecurity Access Scale (HFIAS), 3) Insomnia Scale (ISI), 4) Physical Activity Questionnaires (I-PAQS), 5) The Connor-Davidson Resilience Scale (CD-RISC), 6) Fear of COVID-19 Scale, 7) Post-Traumatic Stress Disorder Scale (PCL-C).

In the general data, we obtained information about age in years, gender (male, female), civil status: single (no, yes), religion (none, Catholic, Non-Catholic), previous disorders (hypertension, diabetes), report of frequent alcohol consumption and smoking, self-reported weight and height, previous personal and family history of a mental disorder, mental health support during the pandemic, trust in the government to manage the pandemic, and time of service on the frontline of defense against COVID-19 pandemic in the military site (1 to 6 months, 7 to 12 months, 13 to 18 months, 19 months or more).

#### Dependent variable

Post-traumatic stress disorder (PCL-C): PTSD symptoms were assessed using the Posttraumatic Stress Disorder Checklist – Civilian Version (PCL-C), a validated 17-item self-report instrument based on DSM-IV diagnostic criteria. Each item is scored on a 5-point Likert scale (1 = not at all to 5 = extremely), yielding a total score between 17 and 85. A cut-off score of ≥44 was applied to classify probable PTSD (46), following international studies in Spanish-speaking populations and prior applications in military and healthcare settings. Although this threshold has not yet been locally validated in Peruvian military personnel, it has demonstrated good diagnostic accuracy in other populations and was selected to ensure comparability with existing literature. Although no structured clinical interview (e.g., CAPS-5) was conducted, the PCL-C has demonstrated strong psychometric properties (internal consistency α = 0.94; test–retest reliability r = 0.82) (47) and is widely used in epidemiological studies where clinical confirmation is not feasible.

#### Independent variables

Household Food Insecurity Access Scale (HFIAS): It was developed by the US Agency for International Development. It includes nine items, which correspond to questions about the food consumed in the last four weeks. The questions ask whether having food insecurity, anxiety, quality and consumption of enough food, and physical consequences (48). The questions are categorized as follows: food security (Question 1), mild food insecurity (questions 2-4), moderate food insecurity (questions 5 or 6), and severe food insecurity (questions 6 - 9) (49). The instrument showed high internal consistency (α = 0.74) (50).

Insomnia scale (ISI): It includes seven items that evaluate the nature, severity, and impact of insomnia. It has been validated in older adults, in primary health care patients, and in Spanish-speaking general population. This instrument showed a reliability coefficient of 0,82 through the Cronbach’s alpha (51). The ISI has shown solid psychometric properties, including high internal consistency (α = 0.91) and convergent validity in Spanish older adults (52), factorial validity in Spanish-speaking medical students (51), and construct/diagnostic validity in U.S. veterans with traumatic brain injury (sensitivity 0.81, specificity 0.71) (53), as well as other clinical populations (54, 55). Insomnia presence was valued with a score superior to eight points (53, 56, 57).

Physical activity Questionnaires (IPAQ-S): It considers the four components of physical activity (leisure, house maintenance, work, and transportation) (58). It has nine items and evaluates physical activity declared in the last seven days. This instrument allows a weighted estimation of physical activity declared by week. The physical activity level is classified as low, moderate, and high. It has been validated in Spanish-speaking populations and has been administered to Latin American population (59).

The Connor-Davidson Resilience Scale (abbreviated version of CD-RISC): This questionnaire is composed of ten questions. It has a Cronbach’s alpha coefficient of 0,89 in general population and a test-retest reliability of 0,87 in people with post-traumatic stress disorder (PTSD) (60). It was evaluated through a five-point Likert scale. A cut-off point of 30 was used to categorize resilience as high (>30) and low (<30) (61). It has been validated in military populations, showing excellent psychometric properties, including internal consistency (α >0.93), test–retest reliability (ICC = 0.88), and measurement invariance across rank, gender, and time in Chinese military personnel (62), as well as good validity and reliability in male soldiers with and without PTSD (63). In Latin America, it has been validated in Peruvian university students, demonstrating unidimensional structure, acceptable reliability (ω > 0.85), and measurement invariance by sex (64). Likewise, evidence from Colombian university populations confirmed adequate factorial validity and reliability of the scale (65). The cut-off point of ≥30 for high resilience on the CD-RISC-10 was selected based on its use in Peruvian studies during the COVID-19 pandemic (66, 67), including research with healthcare workers (61). Although the scale has no universal threshold, categorizations in international studies (e.g., terciles or quartiles) also support interpreting scores ≥30 as indicative of high resilience (68, 69).

Fear of COVID-19 scale: It is a reliable instrument with seven items to assess fear of COVID-19 among general population. This instrument has a Cronbach’s alpha coefficient of 0,82 (70). In the present study we used a higher score than 16,5 as a cut-off point to assume the presence of fear of COVID-19 (71). Additionally, cut-off scores have been proposed in other populations, such as 17.5 in the Arabic version validated among the Syrian general population (α = 0.89) (72) and 17.5 in Chinese university students, where the scale showed good reliability (α = 0.87) and diagnostic accuracy (AUC = 0.70) (73). A study of the psychometric properties of the Spanish version of the Fear of COVID-19 scale in a sample of Peruvian population showed adequate measure properties in terms of reliability and validity (74). In Latin America, the FCV-19S has been validated in different populations. In Peru, it showed good internal consistency (Cronbach’s α = 0.83) and unidimensional structure in university students (75). In Colombia, psychometric evaluations in physicians revealed limitations in the original seven-item version but acceptable internal consistency in the five-item version (ω = 0.68) (76).

### Procedures

We conducted *face-to-face interviews* using self-administered questionnaires in order to obtain sociodemographic, psychosocial, physical and mental health information to measure post-traumatic stress disorder (PTSD).

A field team was formed to conduct the research in the military site in Lambayeque, Peru, during the context of the COVID-19 health emergency. We requested authorization to the officer in charge of the military site. Then, the questionnaire was designed online in the data entry system REDCap. Subsequently, the field team coordinated the interviews in three groups during two different shifts (morning and afternoon). The main researcher explained the objective of the research and invited the military personnel to participate in the study through filling out the questionnaire. This happened while they were in ventilated environments, complying with biosecurity measures (correct use of masks, social distancing, and hand washing). Immediately, the main researcher sent the questionnaire link online to the supervisors of the shift. The supervisors sent the questionnaires to the different WhatsApp groups of coordination; thus, we ensured that the link was shared to all the study population. The first part of the questionnaire contains the informed consent; once the participant accepted to participate, he/she was automatically directed to the self-administered questionnaires of the variables of interest. The average time to fill out the questionnaire was 20 minutes.

### Analysis plan

We exported the Excel database from the REDcap system. Then, we carried out a process of cleaning of the database to identify missing data and/or inconsistent or out of rank data.

The statistical analysis was performed in the STATA v.17.0 program (StataCorp, TX, USA).

In the univariate analysis, we present the best measures of central tendency and dispersion of the numerical variables, after the evaluation of normal distribution. The categorical variables were shown as absolute and relative frequencies.

To compare numerical variables and the outcome of interest (PTSD), we used the Mann- Whitney U test, after evaluating the normal distribution assumption. To compare categorical variables and PTSD, we used the Chi-squared test after having evaluated the assumption of expected frequencies previously.

To evaluate the factors associated with PTSD, we calculated prevalence ratios (PR) and confidence intervals at 95%, using simple and multiple regression models. In the multiple models, the variables that were statistically significant (p<0.05) in the simple model were included. Additionally, multicollinearity was evaluated in the variables of interest. PTSD symptoms (e.g., re-experiencing, avoidance, hyperarousal) were measured using the PCL-C instrument, while smoking, insomnia, and fear of COVID-19 were treated as independent variables rather than components of PTSD.

Multiple regression results for factors associated with PTSD were summarized using a forest plot constructed in RStudio.

### Ethical aspects

The protocol study was approved by the Ethics Committee of the Universidad San Martin de Porres (File N°269-2021-CIEI-FMH-USMP). All participating military personnel provided their informed consent prior to completing the survey, which was anonymous, and confidentiality of the respondents was guaranteed. We respected the ethical principles of the Helsinki Declaration.

## Results

### General characteristics of military personnel

Of the 615 military members, most of them were male (93.7%) and were between 19 and 32 years old. In addition, 73.3% were single; 27.5% had children; 17.4% reported that they consumed alcohol frequently; and 9.1% informed they had hypertension. The majority had a normal body mass index (57.5%), and almost the third part were overweight (34.5%). Moreover, 48.6% experienced food insecurity while 18.4% suffered from subclinical insomnia, and 43.2% showed high resilience (**Table 1**).

**Table 1:** Participant characteristics (n=615).

Characteristics	N (%)
Age (years)*	22 (19-32)
Gender	
Female	39 (6.3)
Male	576 (93.7)
Marital status	
Single	451 (73.3)
Married	143 (23.3)
Cohabiting	13 (2.1)
Divorced	8 (1.3)
Religion	
None	90 (14.6)
Catholic	430 (69.9)
Non-Catholic	95 (15.5)
Having children	169 (27.5)
Alcoholism	107 (17.4)
Smoking	40 (6.5)
Comorbidity	
Hypertension	56 (9.1)
Diabetes	11 (1.8)
BMI (categorized)	
Underweight	9 (1.3)
Normal	400 (57.7)
Overweight	239 (34.5)
Obesity Type 1	39 (5.6)
Obesity Type 2	5 (0.7)
Obesity Type 3	1 (0.1)
Personal mental health history	7 (1.1)
Family mental health history	26 (4.2)
Mental health support	49 (8.0)
Trust in the government to manage COVID-19	
Yes	334 (54.3)
No	281 (45.7)
Time of service	
1 to 6 months	154 (25.6)
7 to 12 months	100 (16.6)
13 to18 months	128 (21.3)
19 months or more	219 (36.4)
Food insecurity	
No	316 (51.4)
Yes	299 (48.6)
Insomnia	
Absence of clinical insomnia	436 (77.0)
Subclinical insomnia	104 (18.4)
Moderate clinical insomnia	17 (3.0)
Severe clinical insomnia	9 (1.6)
Level of physical activity	
low	64 (10.4)
moderate	39 (6.3)
High	512 (83.3)
Resilience	
low	333 (56.8)
high	253 (43.2)
Fear of COVID-19	
No	424 (80.8)
Yes	101 (19.2)
Post-traumatic stress disorder	
No	567 (92.2)
Yes	48 (7.8)

*Median (25th percentile - 75th percentile).

### Post-traumatic stress disorder - PCL-C

**Figure 1:**
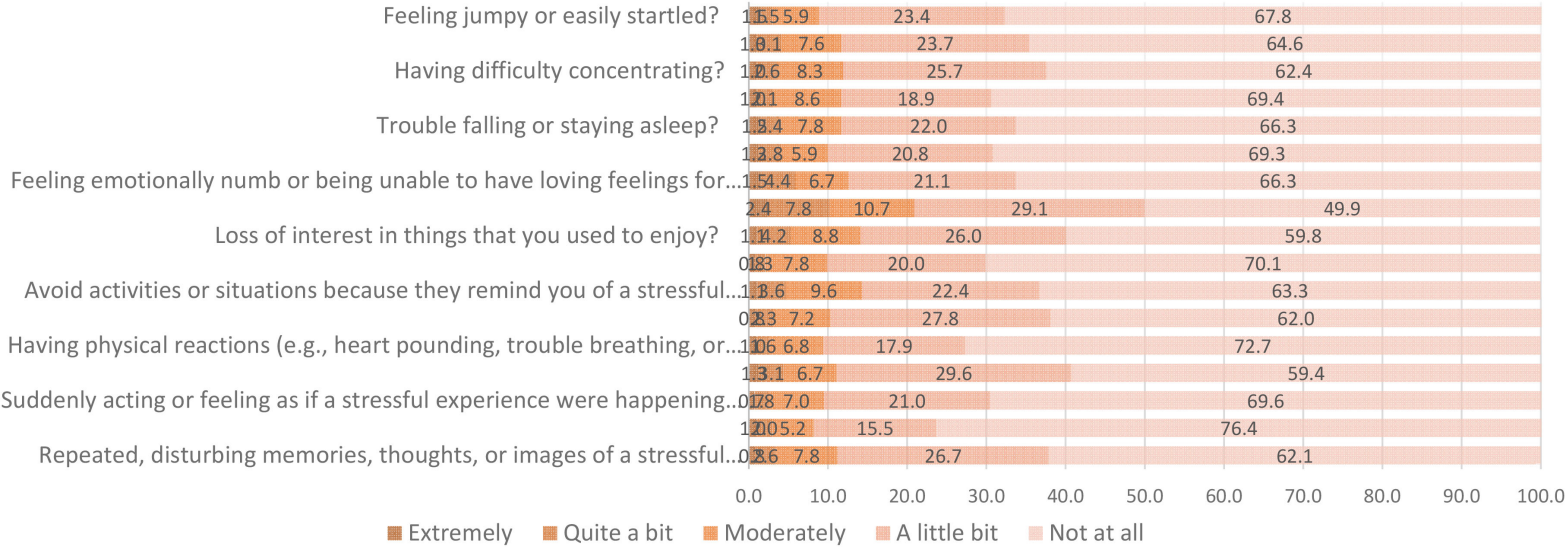
PCL-C Instrument in military personnel.

The prevalence of PTSD was 7.8% (95% CI: 5.8%–10.2%). Of the total, 10.7% mentioned that they felt moderately distant from other people. In addition, 9.6% informed that they avoided moderately activities that remind them of the stressful experience of COVID-19. Finally, 7.8% reported moderately having memories and/or thoughts that occur repeatedly due to past stressful experiences (**Figure 1**).

### Factors associated with post-traumatic stress disorder in the bivariate analysis

The military personnel with fear of COVID-19 had a higher frequency of PTSD, in comparison with those who did not informed fear (29.7% vs 2.1%; p<0.001). PTSD frequency was superior in military personnel with a low level of resilience (9.9% vs. 4.4%; p=0.011), in comparison with military members with a high level of resilience. The military personnel with severe insomnia had a higher frequency of PTSD, in comparison with those without insomnia (55.6% vs 2.1%; p<0.001). Additionally, we found that alcoholism (p=0.025), smoking (p<0.001), previous history of mental health (p=0.039), trust in the government to manage the pandemic (p=0.033), level of physical activity (p=0.003), and time of service (p<0.001) were factors significantly associated with PTSD (**Table 2**).

**Table 2:** Characteristics associated with post-traumatic stress disorder in the bivariate analysis.

Variables	Post-traumatic stress disorder		p*
	No (n=567)	Yes (n=48)	
	n (%)	n (%)	
Age (years)***	22 (19-32)	23.5 (20-29)	0.281**
Male sex	531 (92.2)	45 (7.8)	0.978
Having children	158 (93.5)	11 (6.5)	0.461
Alcoholism	93 (86.9)	14 (13.1)	**0.025**
Smoking	29 (72.5)	11 (27.5)	**<0.001**
Comorbidity			
Hypertension*	51 (91.1)	5 (8.9)	0.742
Diabetes	10 (90.9)	1 (9.1)	0.873
BMI (categorized)			0.748
underweight/normal	332 (92.2)	28 (7.8)	
Overweight	186 (91.6)	17 (8.4)	
Obesity	39 (95.1)	2 (4.9)	
Personal mental health history (Yes)	5 (71.4)	2 (28.6)	**0.039**
Family mental health history (Yes)	24 (92.3)	2 (7.7)	0.983
Mental health support (Yes)	43 (87.8)	6 (12.2)	0.227
Trust in the government to manage COVID-19 (No)	252 (89.7)	29 (10.3)	**0.033**
Time of service			**<0.001**
1 to 6 months	148 (96.1)	6 (3.9)	
7 to 12 months	88 (88.0)	12 (12.0)	
13 to18 months	107 (83.6)	21 (16.4)	
19 months or more	211 (96.4)	8 (3.7)	
Food insecurity (Yes)	272 (91.0)	27 (9.0)	0.271
Insomnia			**<0.001**
Absence of clinical insomnia	427 (97.9)	9 (2.1)	
Subclinical insomnia	82 (78.9)	22 (21.2)	
Moderate clinical insomnia	11 (64.7)	6 (35.3)	
Severe clinical insomnia	4 (44.4)	5 (55.6)	
Level of physical activity			**0.003**
Low	52 (81.3)	12 (18.8)	
Moderate	36 (92.3)	3 (7.7)	
High	479 (93.6)	33 (6.5)	
Resilience (High)	242 (95.7)	11 (4.4)	**0.011**
Fear of COVID-19 (Yes)	71 (70.3)	30 (29.7)	**<0.001**

*p-value of categorical variables calculated with the Chi-squared test.

**p-value of categorical-numerical variables calculated with the U test (Mann-Whitney).

***Median - interquartile range.

### Factors associated with post-traumatic stress disorder in the simple and multiple regression model analysis

Through simple regression analysis, we could find that the factors associated with a higher prevalence of PTSD were alcoholism (PR: 1.95; 95% IC: 1.09-3.52), smoking (PR: 4.27; 95% IC: 2.18-8.38), history of mental health (PR: 3.78; 95% IC: 1.13-12.60), trust in the government to manage the pandemic (PR: 1.81; 95% IC: 1.04-3.17), having been working between 13 and 18 months (PR: 4.21; 95% IC: 1.75-10.12), insomnia (PR: 12.30; 95% IC: 6.04-25.04) fear of COVID-19 (PR: 13.99; 95% IC: 6.86-28.56). On the contrary, the factors associated with a lower level of prevalence of PTSD were the following: having a high level of physical activity (PR: 0.34; 95% IC: 0.19-0.63), and a high level of resilience (PR: 0.44; 95% IC: 0.23-0.85) (**Table 3**).

**Table 3:** Factors associated with post-traumatic stress disorder in Lambayeque, in the analysis of simple regression model.

Characteristics	Simple regression		
	PR	95% CI	p*
Age (years)	1.00	0.97-1.02	0.810
Gender			
Female	Ref.		
Male	1.02	0.33-3.12	0.978
Marital status			
Single	Ref.		
Married	0.46	0.20-1.07	0.070
Cohabiting	0.85	0.13-5.70	0.864
Divorced			
Religion			
None	Ref.		
Catholic	1.83	0.67-5.03	0.240
Non-Catholic	2.13	0.68-6.68	0.194
Having children	0.78	0.41-1.50	0.464
Alcoholism	1.95	1.09-3.52	**0.025**
Smoking	4.27	2.18-8.38	**<0.001**
Comorbidity			
Hypertension	1.16	0.48-2.81	0.741
Diabetes	1.17	0.18-7.74	0.872
BMI (categorized)			
underweight/normal	Ref.		
Overweight	1.07	0.60-1.92	0.802
Obesity	0.63	0.15-2.54	0.513
Personal mental health history			
No	Ref.		
Yes	3.78	1.13-12.60	**0.031**
Family mental health history			
No	Ref.		
Yes	0.98	0.25-3.84	0.983
Mental health support			
No	Ref.		
Yes	1.65	0.74-3.69	0.223
Trust in the government to manage COVID-19			
Yes	Ref.		
No	1.81	1.04-3.17	**0.036**
Time of service			
1 to 6 months	Ref.		
7 to 12 months	3.08	1.19-7.95	**0.020**
13 to18 months	4.21	1.75-10.12	**0.001**
19 months or more	0.94	0.33-2.65	0.903
Food insecurity			
No	Ref.		
Yes	1.36	0.79-2.35	0.273
Insomnia			
No	Ref.		
Yes	12.30	6.04-25.04	**<0.001**
Level of physical activity			
Low	Ref.		
Moderate	0.41	0.12-1.36	0.146
High	0.34	0.19-0.63	**0.001**
Resilience			
Low	Ref.		
High	0.44	0.23-0.85	**0.015**
Fear of COVID-19			
No	Ref.		
Yes	13.99	6.86-28.56	**<0.001**

**Figure 2:**
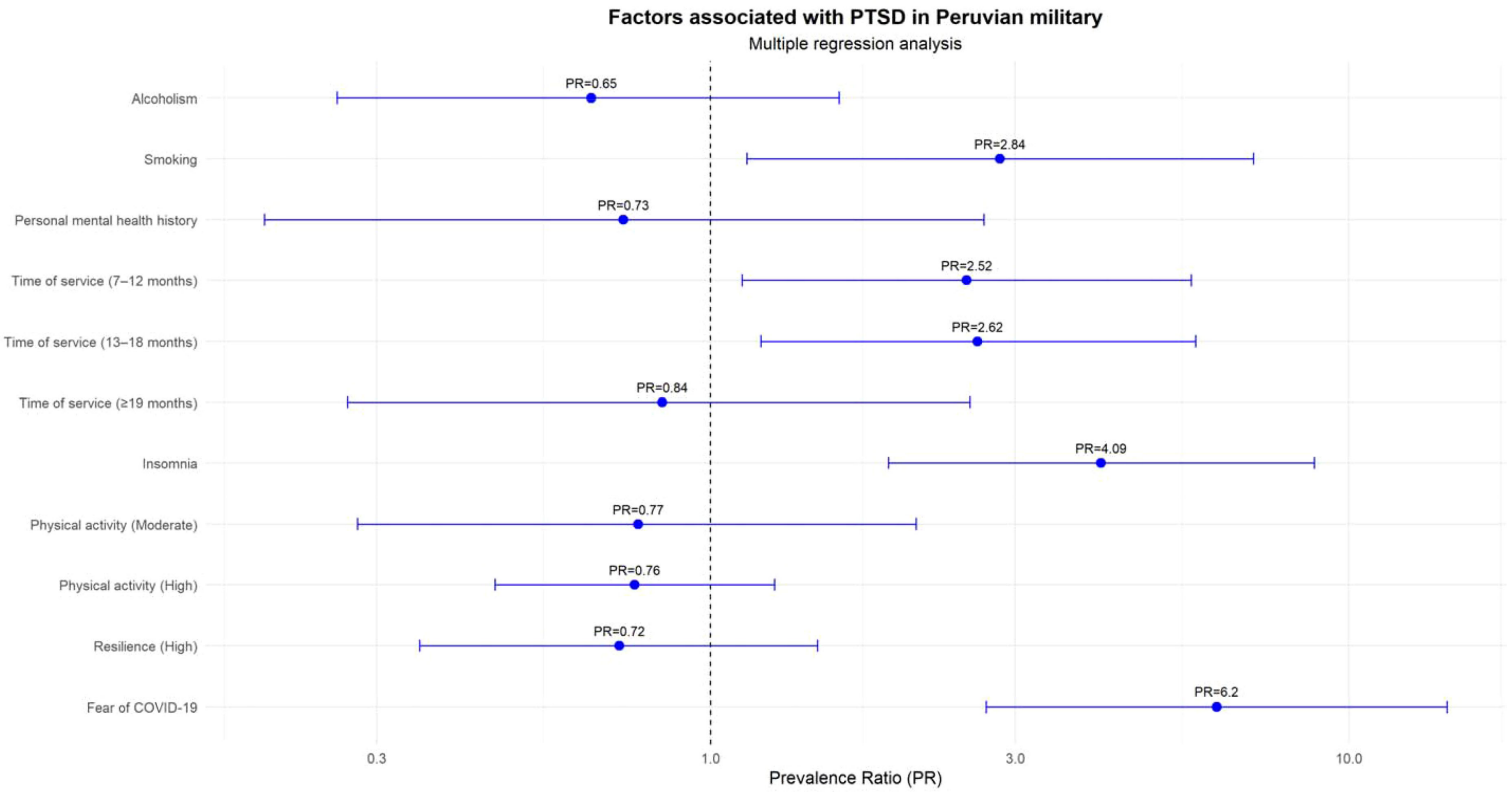
Factors associated with PTSD in military personnel.

The analysis of the multiple regression model showed that smoking (PR: 2.84; 95% IC: 1.14-7.09), having between seven and 12 months of service (PR: 2.52; 95% IC: 1.12-5.66), and from 13 to 18 months (PR: 2.62; 95% IC: 1.20-5.75) were associated with a higher level of prevalence of PTSD in military personnel. Military members with insomnia were associated with a 309% higher prevalence of PTSD (PR: 4.09; 95% IC: 1.90-8.83). Likewise, experiencing fear of COVID-19 was associated with a 520% higher prevalence of PTSD (PR: 6.20; 95% IC: 2.70-14.22), compared with those who did not report this fear (**Figure 2**).

### Correlation analysis

PCL-C scores were positively correlated with insomnia severity (ρ = 0.55, p < 0.001) and fear of COVID-19 (ρ = 0.50, p < 0.001), while a weak negative correlation was observed with resilience (ρ = –0.11, p = 0.011). **Table 4**.

**Table 4:** Spearman’s correlations between PCL-C scores and key variables.

Variable	ρ	P-value
Insomnia severity (ISI)	0.55	**<0.001**
Resilience (CD-RISC-10)	–0.11	**0.011**
Fear of COVID-19 (FCV-19S)	0.5	**<0.001**

ρ = Spearman’s rank correlation coefficient.

## Discussion

### Prevalence of post-traumatic stress disorder

As far as we know, this is the first study that assesses PTSD in a representative sample of military personnel from a Latin American country. We found that almost one out of ten participants (7.8%) had PTSD. This prevalence is lower than that reported by Corzo P. et al. (77), who reported a prevalence of 16.6% in Colombian combatants. It is important to highlight that these authors used a different instrument (CAPS) and a smaller sample (42 participants). Similarly, Almanza Muñoz et al. (23), in Mexico, estimated a prevalence of 4.3% in the Mexican army. We can remark that this is the only study conducted in Latin America. Kaspersen y Matthiesen (78) reported a prevalence of 2.8 to 7.8% among the Norwegian soldiers. Marmar et al. (39) reported a prevalence of 4.5% and 11.2% in Vietnamese military members. We have to mention that these studies were conducted in pre-pandemic contexts and used different instruments (psychiatric interview, PSTSS-10, and M-PSTD). The results were supported by the meta-analysis carried out by Petereit-Haack et al. (79), who found a prevalence of PTSD of 1.67% after analyzing 22 studies that used PCL-C as an evaluation instrument.

In contrast, studies conducted during the COVID-19 pandemic have generally reported higher prevalence levels. Hill et al., who reported a prevalence of 16.6% of PTSD among American military personnel (18 to 44 years old) (15). Similarly, Pedersen et al. reported that 31.2% of combat veterans had PTSD (26). Pietrzak et al. reported that 12.8% of military members had PTSD symptoms (27). We can note that these studies were conducted in the first wave of the pandemic and used the PCL-5 instrument. Our results are different from the ones found in studies among health personnel on the first line of defense with a prevalence of 10.7 to 19.9%, in which the Chinese version of the Essen Trauma Inventory (ETI) and PCL-5 were used as measurement instruments, respectively (80, 81). Similarly, in the general population, prevalence values of 4.6 to 55.3% have been reported, in which the Acute Stress Disorder Scale instrument was used (82, 83). We can highlight that these studies were not conducted in the first wave and in populations who are not Latin American. Therefore, our results contribute to the literature about this topic and with this population group in particular, as they may be more vulnerable to developing mental health disorders such as PTSD. This vulnerability may be related to factors such as sleep quality disturbances (84), isolation and family distancing (85) that were more frequently reported during the pandemic.

### Factors associated with post-traumatic stress

Through this research, we found that a greater number of months working on the frontline of the COVID-19 health emergency was associated with a higher prevalence of PTSD, as participants who had between seven and 12 months and between 13 and 18 months of service showed 152% and 162% higher prevalence, respectively. This is similar to what was reported by Gonzáles-Penagos et al. (86) who showed that the participants with the highest working time (eight and 12 hours a day) had higher levels of prevalence (PR = 4,24; 95% IC, 0,71-25,3) of PTSD in Colombian forces who are exposed to public order disturbances. Similarly, Grieger et al. (87) identified that the active American soldiers had PTSD at the 1rst, 4th, and 7th month (4.4%, 12.2%, and 12.0%, respectively). In addition, Grieger et. al (13)., in their meta-analysis of 32 studies, showed that a greater number of time of service is directly correlated with the increase in the probability of developing PTSD (OR = 1,28; 95% IC, 1,01-1,26) in active military personnel and veterans. We can highlight that these three studies were not conducted in the pandemic context for which our result contributes with evidence to current literature. Those findings may be understood in light of the fact that time of service, deployment, and the re-experiencing of active duty tend to be associated with higher levels of PTSD symptoms. These associations may have been further reinforced by the constant stress experienced during the pandemic (the study population corresponded to war veterans) (88).

Participants who reported frequent smoking were associated with a 184% higher prevalence of PTSD. Similarly, the study conducted by Currie Cheryl in Canadian general population in the pandemic context found that females and males that consumed substances were 2.2 and 2.3 times more likely to have post-traumatic stress symptoms, respectively (89). This is similar to what was reported by Dabs et al. (90), who showed that active military personnel with substance dependency (opiates) had higher probability (OR = 28; CI: 21,2-37,7) of developing post-traumatic stress. In addition, Cotter LB et al. (91) showed a positively significant association between substance use (cocaine/opiates) and PTSD symptoms in general population. Moreover, it has been noted that the onset of substance use occurs in parallel with PTSD symptoms (92). We can highlight that the data reported in these three studies come from non-pandemic contexts. Models of association between these variables have been proposed, in a bidirectional manner, whereby patients with PTSD may use substances as a coping strategy to alleviate symptoms, while substance use itself may be related to an increased likelihood of experiencing PTSD symptoms (68, 71), as in our study. Among the proposed mechanisms is “self-medication” referring to people with PTSD who smoke cigarettes in order to alleviate and cope with the symptoms of the disorder (72). However, the number of studies examining these associations remains limited (90–95).

Similarly, those who had insomnia showed an association with a 309% higher prevalence of PTSD. This result is consistent with a study that was conducted in people infected with COVID-19 in the United States, which showed that the relationship between the long-term variable increased significantly in a positive direction (96). In addition, a study carried out in the pandemic context in general population that evidenced insomnia as a risk factor for PTSD according to the time since the start of the pandemic, which was a 26% higher after four months and 40.0% after nine months, different from those who did not have insomnia (97). This is similar to what was reported by McLay et al. (98), who reported insomnia as the main comorbidity that is associated with developing PTSD among military members returning from deployment activities. Armenta et al. (10) identified that military personnel that slept less than four hours a night had a higher risk of developing persistent PTSD. Miles et al. (99) demonstrated the PTSD severity was associated with severity of nightmares, sleep time, and insomnia. It is important to highlight that these studies were performed in pre-pandemic contexts. The findings could be explained by the fact that having comorbidity conditions lead to a dysregulation of the neuroendocrine system (100), where the exact mechanisms are still unknown in the context of the pandemic (101). Furthermore, it has been postulated that insomnia is a predictor of long-term PTSD; however, the bidirectional relationship between the variables is highlighted (10). Finally, it has been proved that the Mindfulness-Based Cognitive Therapy focused on sleep (MBCT) reduces PTSD symptoms (102).

Fear of COVID-19 was strongly associated with a 520% higher prevalence of PTSD. This is similar to what was reported by McLay et al. (103), who proved that those American military veterans were concerned about the pandemic were more likely to have PTSD (OR = 1,10, 95% IC:1.05-1.14). Similarly, Hendrikx et al., during the pandemic, reported that those military veterans who were more worried as a stressor increased the probability of PTSD (OR = 6,30, 95% IC:1.97-20.13). We can mention that they used the PCL-5 instrument to measure PTSD and did not use a validated instrument to measure fear of COVID-19. On the other hand, they formulated a variety of questions related with the effects of the pandemic (104). Supporting our result, it has been identified that there exists a relationship between fear of COVID-19 and the development of mental health disorders (PTSD, depression, anxiety, and stress) in Canada, Italy and Peru (34–36). However, we did not find studies that evaluate the same variables in military population; thus, our study contributes to the existent literature. This association may be related to fear of death from COVID-19 or the loss of a relative, which can constitute traumatic experiences and intense grief, and both situations may act as potential risk factors for chronic PTSD symptoms (105).

Finally, the prevalence of PTSD appeared to be reduced by 56% in participants with high resilience; however, this protective association was not statistically significant in the final adjusted model. This is similar to what was reported by de Roon-Cassini et al. (106) who identified that people who reported high resilience did not present PSTD symptoms. Moreover, Ghaffarzadegan et al. (107) suggested that resilience may be effective in mitigating PTSD symptoms in military personnel and veterans. One possible explanation for the lack of statistical significance in our final model is that resilience may overlap with other proximal determinants such as insomnia and fear of COVID-19, which exert stronger direct associations with PTSD. In this sense, resilience could act as a distal coping factor whose apparent protective role becomes attenuated once acute stressors and comorbidities are accounted for. Another explanation may involve residual confounding, as unmeasured psychosocial resources (e.g., social support, prior trauma exposure) were not included in our models. This potential effect may be related to adaptive coping and positive personality traits that are associated with a lower frequency of PTSD symptoms (104). It should be noted that the studies were conducted in a pre-pandemic context. Overall, our findings suggest that resilience alone may not be sufficient to buffer PTSD risk in high-intensity contexts such as the pandemic, highlighting the need for integrated interventions that combine resilience-building strategies with approaches targeting sleep health, stress management, and broader psychosocial support. One interpretation of this finding is that military training and the acquisition of adaptive coping skills in the management of previous stressors could help enhance resilience in regard to pandemic-related stressors (108). Although resilience was analyzed as a covariate in our models, it may also act as a moderator, influencing how stressors such as insomnia or fear of COVID-19 translate into PTSD symptoms. Future studies with longitudinal designs are needed to examine these potential interaction effects. Furthermore, while we applied the ≥30 threshold to ensure comparability with local literature, alternative approaches such as tertile- or percentile-based classifications could yield different patterns and should be considered in future research.

In our study, smoking, insomnia, and fear of COVID-19 emerged as factors associated with higher PTSD prevalence. These should not be interpreted as PTSD symptoms themselves. PTSD symptoms—such as flashbacks, avoidance, and hyperarousal—were assessed directly through the PCL-C instrument. Our findings therefore highlight how external behavioral and psychosocial exposures can increase vulnerability to PTSD, beyond the disorder’s intrinsic symptomatology.

### Implications of findings for mental health

Our results show that PSTD is prevalent in military populations. Post-pandemic interventions should focus on detection and treatment in order to mitigate the psychiatric sequelae of an unanticipated event and prevent the occurrence of persistent PTSD. Given that there is a high level of prevalence in active military personnel, screening methods become a public health necessity, such as those carried out in our country through the directives of the Ministry of Health. These directives allocate a monthly budget for mental health screening (109) and the Peruvian army, which implements a comprehensive health care model for the military community at the primary health care level (110). In addition, our results could serve as a precedent for having been carried out in Peruvian military personnel, which adds Latin American evidence to the world literature.

From a policy perspective, our findings support the need for annual screening of PTSD symptoms among active-duty military personnel, with particular attention to individuals with insomnia, smoking habits, or elevated fear of infectious threats. Referral pathways to mental health professionals should be standardized, ensuring timely access to specialized care. At the unit level, insomnia reduction and sleep-hygiene programs could be implemented to address one of the strongest predictors identified in our analysis. Furthermore, resilience-building interventions (e.g., stress management workshops, peer support groups) should be incorporated into ongoing military training, strengthening protective factors against PTSD in future crises.

### Limitations and strengths

Our findings are subject to several limitations that should be considered. First, the cross-sectional design precludes establishing causality between independent variables and PTSD. As a result, the associations identified should not be interpreted as causal effects, and it remains uncertain whether factors such as smoking, insomnia, and fear of COVID-19 preceded the development of PTSD or emerged as consequences of it. We also emphasize that neither correlations nor prevalence ratios establish causality, and future longitudinal cohort or case–control studies are warranted to confirm temporal relationships. Nevertheless, we used validated instruments with strong internal consistency, such as the PCL-C, which strengthens measurement validity (111). Second, the use of self-administered questionnaires may have introduced recall or reporting bias. To minimize this risk, participants were recruited during active service and data collection was coordinated in person, ensuring comprehension of the questions. Third, limitation relates to the sampling strategy. Because snowball sampling was used, there is potential for selection bias, which may have affected the representativeness of the sample and limited the external validity of the findings. Although this approach was the only feasible strategy during the COVID-19 state of emergency, efforts were made to reduce bias by coordinating recruitment through unit supervisors, achieving a high participation rate (86.6%), and including personnel from all service groups within the brigade. Fourth, unmeasured confounders (e.g., previous trauma exposure, social support, coping strategies) were not captured in our models, which may have influenced the observed associations. Another limitation is that the psychometric instruments used (ISI, CD-RISC-10, and FCV-19S) have not been specifically validated in Peruvian military populations. However, they have demonstrated robust validity and reliability in culturally and contextually relevant groups, such as Spanish- and Latin American university students, healthcare workers, and military samples in other countries, including during pandemic contexts (2, 66, 84, 112). This supports their applicability to our study population, although further validation in Peruvian military personnel remains warranted. Also, PTSD was assessed using the PCL-C with a cut-off of ≥44 points. While this threshold is widely used and has been validated in other Spanish-speaking and military populations (67), it has not been specifically validated in Peruvian military personnel, which could have led to a slight misclassification of cases. Furthermore, our findings may not be generalizable to other military populations or regions, as cultural norms, operational structures, and context-specific stressors vary widely across armed forces. Therefore, the results should be interpreted within the context of military personnel from northern Peru, a region heavily impacted by COVID-19 morbidity and mortality during the pandemic, and future research is needed to examine whether similar patterns are observed in other Peruvian or international military settings. Another limitation is that PTSD was assessed through the PCL-C self-report scale rather than a structured clinical interview (e.g., CAPS-5), which may slightly over- or underestimate prevalence; however, the PCL-C has strong psychometric validity and is widely used in epidemiological research. Finally, another limitation is the reliance on self-administered questionnaires, which are vulnerable to information bias, including recall errors and social desirability effects. These biases may have led to underreporting of sensitive behaviors (e.g., smoking, alcohol use) or mental health symptoms. These factors could have affected both the prevalence estimates and the observed associations.

This study presents several strengths. First, it addresses PTSD in military personnel during the COVID-19 pandemic, a critical and underexplored issue in Latin America. Second, the relatively large sample of 615 participants, collected during the second wave when military personnel were actively on duty, enhances statistical power and contextual relevance. Third, we employed well-established psychometric instruments (e.g., PCL-C, ISI, CD-RISC, FCV-19S), many of which have been validated in Spanish-speaking populations, ensuring cultural and linguistic applicability. Fourth, the use of both bivariate and multivariate regression models strengthened the robustness of the analyses.

## Conclusions

It was found that almost one out of ten participants had PTSD. Among the factors associated with PTSD, we can mention number of years of service, smoking, insomnia, and fear of COVID-19. High resilience was associated with lower prevalence in crude models but was not statistically significant after adjustment. The found results highlight the importance of evaluating, following, and treating mental health disorders in active military members.

## Data Availability

The raw data supporting the conclusions of this article will be made available by the authors, without undue reservation.
